# Supplementary figures and images for: Intestine-to-neuronal signaling alters risk-taking behaviors in food-deprived Caenorhabditis elegans
Source: PLoS Genet. 2022 May 5;18(5):e1010178. doi: 10.1371/journal.pgen.1010178 (PMC9070953; doi:10.1371/journal.pgen.1010178)

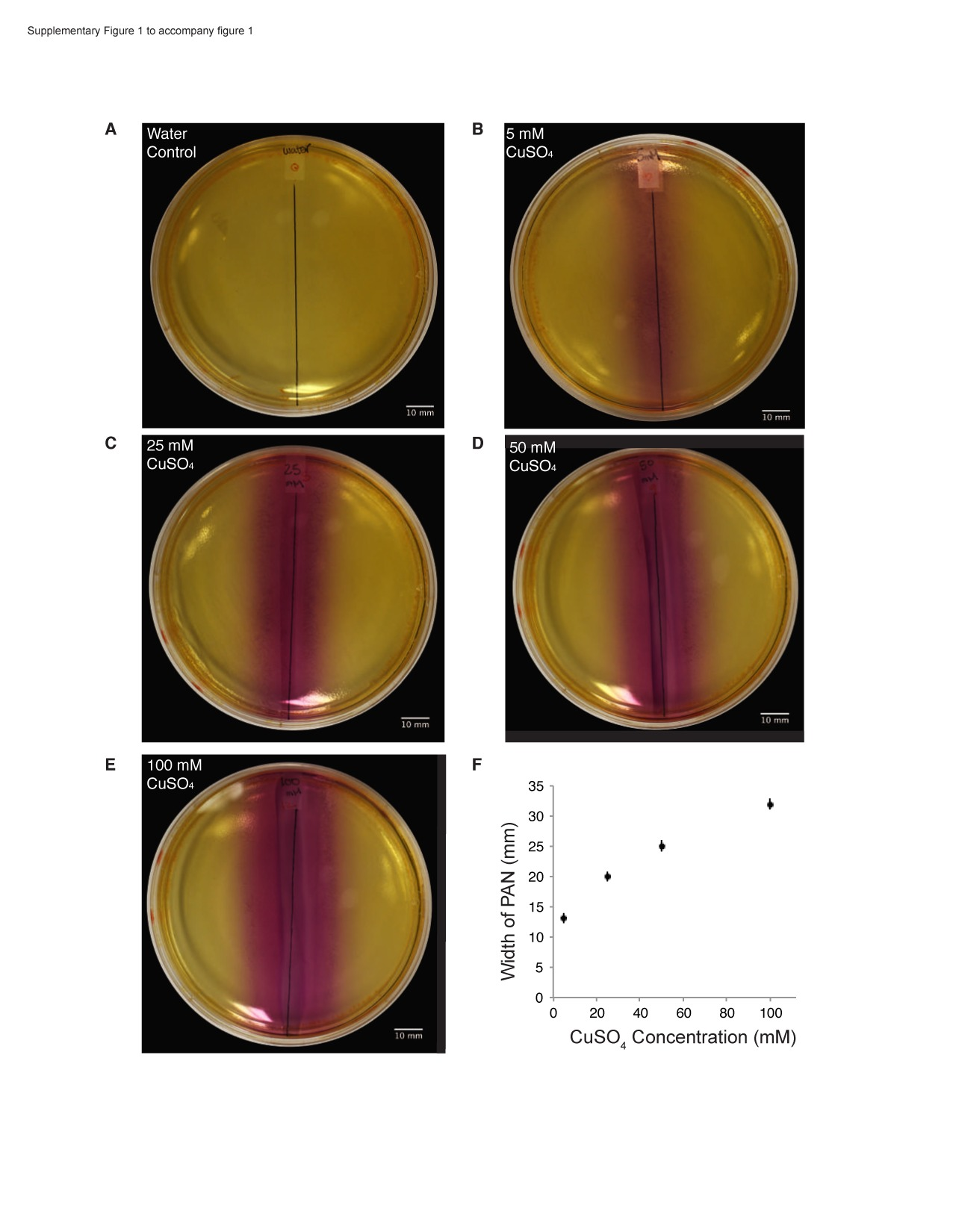

Supplement: S1 Fig (to accompany Fig 1) — (A-E) 25 μl of (A) water as control, (B) 5 mM CuSO4, (C) 25 mM CuSO4, (D) 50 mM CuSO4, and (E) 100 mM CuSO4 was dripped and dried overnight along the midline of the plate to form a copper gradient. PAN indicator (1-(2-pyridylazo)-2-naphthol) distributed over the entire plate shows a gradient of orange-red upon chelation with copper ions. (F) Measured width of colored area with each data point representing the average width, error bars indicate SEM. n = 9. (TIF) [file pgen.1010178.s001.tif]

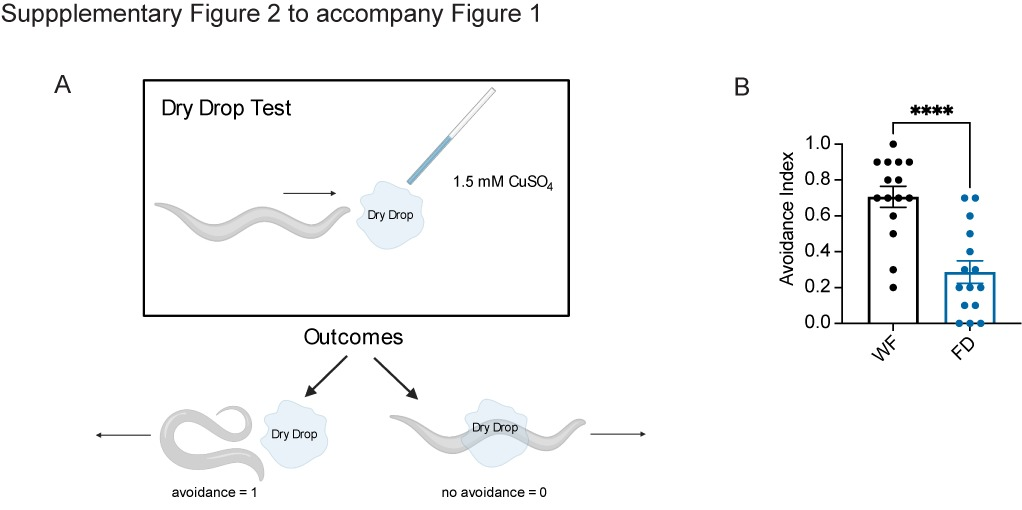

Supplement: S2 Fig (to accompany Fig 1) — (A) Schematic for dry drop test shown in B. ~300 nL of 1.5 mM CuSO4 is dropped ~1 mm away from the animal’s forward motion. Turning away or backing up is considered “avoidance” and given a score of 1. Heading toward the dried drop is considered “no avoidance” and given a score of 0. (B) Quantification of the dry drop test. Food-deprived (FD) animals were starved for 3 hours. Each dot represents the average of ten trials (drops) for a single animal, n = 15. Analyzed with an unpaired t-test * p<0.5, ** p<0.01, *** p<0.001, **** p<0.0001, ns p>0.05. Error bars are S.D. Created with Biorender.com. (TIF) [file pgen.1010178.s002.tif]

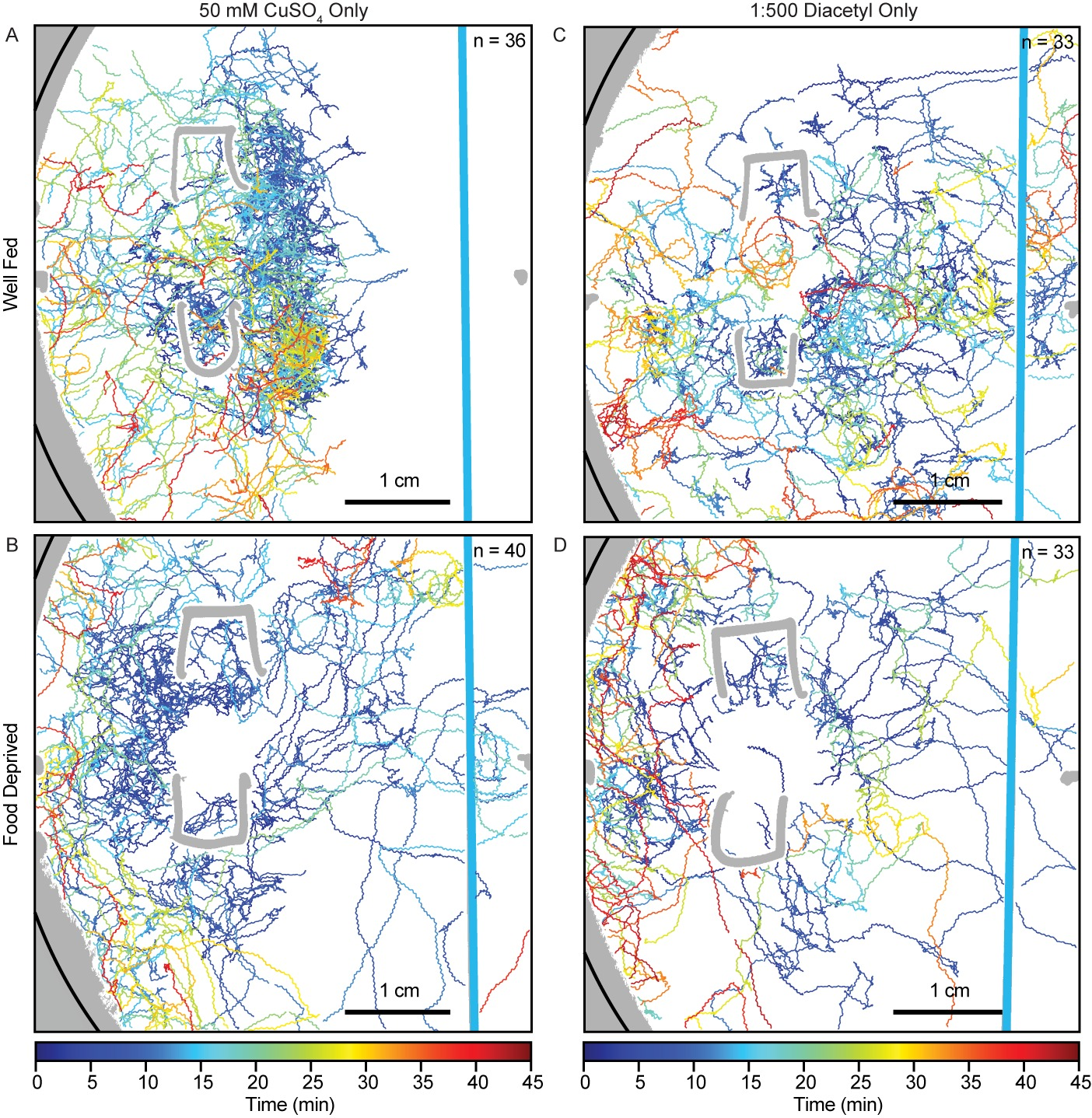

Supplement: S3 Fig (to accompany Fig 2) — (A) Worm tracks (n = 36) are plotted for a representative sensory integration assay of well-fed animals behaving in the presence of 50 mM CuSO4 in water (blue stripe) with no attractant (location out of view to the right). Regions of the plate that were not able to be tracked are in gray with the edge of the plate indicated in black. Tracks are plotted and color coded for time. (B) Worm tracks (n = 40) are plotted for a representative sensory integration assay of 3 hour food-deprived animals. Conditions and plotting the same as in A. (C) Worm tracks (n = 33) are plotted for a representative sensory integration assay of well-fed animals behaving in the presence of no barrier (blue stripe) with attractant is 1 μL 0.2% diacetyl (1:500) in 100% ethanol (location not shown). Plotting the same as in A. (D) Worm tracks (n = 33) are plotted for a representative sensory integration assay of 3 hour food-deprived animals. Conditions the same as in C and plotting the same as in A. (TIF) [file pgen.1010178.s003.tif]

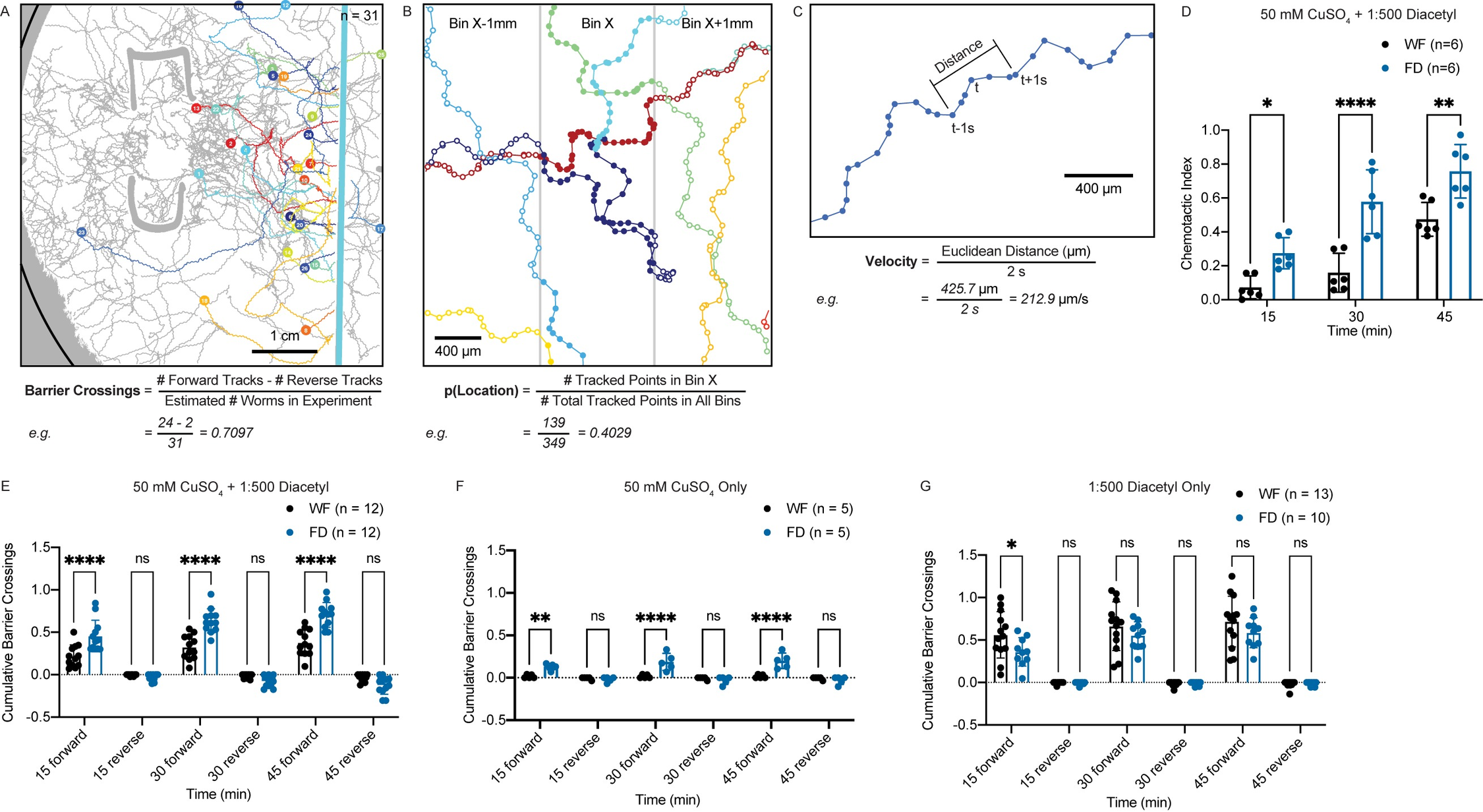

Supplement: S4 Fig (to accompany Fig 2) — (A) Measuring Barrier Crossings. Worm tracks (n = 31) are plotted for the entire 45 minutes of a representative sensory integration assay. 26 tracks that terminate at the copper barrier are plotted in a unique color with the start of each track labelled with a numbered, circular marker. To obtain a measure of barrier crossing, the number of unique, continuous, reverse moving tracks (i.e. tracks that terminate on the right side of the copper barrier) was subtracted from the number of forward moving tracks (i.e. tracks that end on the left side of the copper barrier) and then divided by the estimated number of animals in the experiment. In the example experiment shown, 24 unique forward tracks and 2 reverse tracks were found for the 31 animals assayed, resulting in a Barrier Crossings score of 0.7097 for this experiment after 45 mins of recording. (B) Measuring Probability of Location. 9 unique animal tracks are plotted in a 3 mm x 3 mm field-of-view, a 9 mm2 inset of a 45-minute example experiment. The midpoint positions of the animals at each frame are plotted as circles connected by lines. Midpoints located in Bin X (1 mm wide) are represented by filled circles while midpoints located in the neighboring bins (Bin X-1 and Bin X+1, each 1 mm wide) are represented by open circles. The probability of a worm being located in Bin X is calculated by dividing the number of tracked midpoints in Bin X by the total number of tracked points in all bins. In the small example area shown, there are 139 points in Bin X and a total of 345 points across all 3 bins resulting in a p(Location) score of 0.4029. In the entire field of view there are 45 bins, yielding an average p(Location) score of 0.0222. This analysis was used in Fig 2D, 2G and 2J. (C) Measuring Velocity. 10 seconds (i.e. 30 frames) of a single example worm track is plotted. The midpoint positions of the worm at each frame as identified by WormLab are plotted as filled circles connected by lines. For each time t, the v [file pgen.1010178.s004.tif]
