# Supplementary material for: Intestine-to-neuronal signaling alters risk-taking behaviors in food-deprived Caenorhabditis elegans
Source: PLoS Genet. 2022 May 5;18(5):e1010178. doi: 10.1371/journal.pgen.1010178 (PMC9070953; doi:10.1371/journal.pgen.1010178)
Supplement: Attachment — Submitted filename: Matty_reviewerresponses.docx [file pgen.1010178.s010.docx]

Responses (in black) to the reviewers comments (in blue)

**Reviewer #1:** In this manuscript, the authors studied molecular mechanisms underlying starvation-dependent decreases in avoidance responses toward copper ions using sensory integration assays between copper ions and attractive odorants and quantifying avoidance responses toward copper ions. They found that the mechanical stimuli were not sufficient for the decreased copper ion avoidance responses by analyzing the effects of Sephadex gel beads as tactile stimulation. Next, they used C. elegans mutants of a MondoA ortholog MML-1, a transcription factor regulated by glucose in the cytoplasm, and bHLH transcription factor HLH-30, which was reported to be activated by MML-1. They showed that mutants of MML-1 and HLH-30 showed defects in starvation-dependent decreased copper ion responses. Furthermore, nuclear localization of MML-1 and HLH-30 was altered by the presence of food. On the other hand, mutants of multiple insulin-like genes which were reported to have HLH-30 binding elements, the insulin/IGF-I receptor homolog DAF-2, PI 3-kinase AGE-1 and a TORC2 complex component RICT-1 also showed defects in starvation-dependent decrease in copper ion responses. Using cell-type-specific rescue experiments, they showed that daf-2 and rict-1 functioned in ASI neurons and mml-1 functioned in the intestine in decreased copper ion responses in starvation conditions. From these experimental results, they proposed a model in which MML-1 and HLH-30 in the cytoplasm promote insulin-like peptide release from the intestine in food-deprived conditions, which in turn activates DAF-2/RICT-1 signaling in the ASI neuron to decrease responses toward copper ions.

The manuscript is clearly described and the proposed model is interesting. It is noteworthy that the authors found a new function for mml-1 in behavioral plasticity. However, there are several issues that should be addressed before publication.

We thank the reviewer for their positive feedback. We hope to address the points below.

Major points
1. Although the authors mentioned about some previous researches reporting starvation-dependent behavioral changes (line 75-79, page 4), they did not mention about a previous research, J. Vis. Exp. (125), e55939, doi:10.3791/55939 (2017), showing that avoidance of copper ions was decreased in interaction assay between copper ions and food, which is a similar phenomenon shown in Fig. 1A, B. This paper should be referenced and the authors could revise the manuscript to clearly show the differences from the past findings.

This study (Campbell et al 2017) uses an assay to probe the effects of food-deprivation on repellent sensitivity that is distinct from our current manuscript. These authors use a similar configuration to the assay use in our study, but use a bacterial lawn instead of the diacetyl attractant along with the copper barrier. We are able to test whether different attractants can modify the behavior of food-deprived animals in our assay (which we find that they don’t, however we did not test OP50 as an attractant in our experiments). We do cite this study in our revised manuscript and provide more context. Line 259-263.

2. In a paper, EMBO J 2011 Mar 16;30(6):1110-22, which was referenced, it was shown that avoidance behavior and ASH activities in response to copper ions were decreased after food deprivation. Therefore, a conclusion, “food-deprived animals display reduced sensitivity to copper” (line141-142, page 7), is not a novel finding. Rather, in the current paper, the authors observed only the behavioral responses to copper without seeing neuronal response to copper. So, the current paper does not show experimental evidence showing sensitivity to copper is altered by food deprivation.

We broadly agree with the reviewer. We don’t include any neuronal responses to copper stimuli and have toned-down our conclusions. Line 292-293.

3. In line 212-213, page 10, the authors conclude that “the lack of food in the C. elegans intestine, but not the absence of chemosensory cues, reduces the animals’ sensitivity to copper”. In the experiment in Fig. 3E, F, the authors examined the tactile stimuli but not the chemosensory stimuli.

We include additional data to show that chemosensory cues do not influence the food-deprivation triggered behavioral change in the sensory integration assay. This assay uses agar plugs containing an OP50 lawn attached to the top of the lid of the starvation or well-fed plates. These data show that the odor of food is not sufficient to rescue the food deprivation behavior. These data are provided in Figure 3G and 3H.

4. As mentioned in line 171-172, page 8, the trial number shown in Fig. 2F is smaller than that in Fig. 2C or I. In view of Fig. 1E where chemotaxis with only CuSO4 significantly changes, the lack of significant difference in Fig. 2F is formally a contradiction. To conclude the behavioral mechanisms presented, the authors should repeat the tracking experiment observing copper ion responses on agar plates with only CuSO4 (Fig. 2F, G, H).

We were able to include additional data (N=5) to show that food-deprived animals cross the copper barrier in significant numbers compared to their well-fed counterparts in assays in which there is no attractant. We do note, however, that the proportion of animals crossing is still low and indicates that the use of an attractant makes these assays more robust. These data are presented in updated figures 2F, G, and H. We also included more N for the other conditions in this assay, leading to updated Figures 2C-E and Figures 2I-K. Further, we have simplified and clarified the methods for this tracking in Supplementary Figure S4A, and Supplementary Figure S4E-G.

5. The authors concluded that “MML-1 and HLH-30 accumulation in the cytoplasm (in food deprived animals) enables the intestine to release peptide(s) relaying a “lack of glucose” signal to other tissues” (line 342-344, page 15-16). But the experimental results about HLH-30 localization (Fig. 4G) are difficult to interpret and are not sufficient to support the above conclusion. First, 1hr food deprivation decreased cytoplasmic HLH-30 accumulation (Fig. 4G), whereas it caused decreased copper avoidance (Fig. 1B). The authors should discuss HLH-30 function after 1hr food deprivation. Second, 3hr food deprivation increased cytoplasmic HLH-30 accumulation compared to that after 1hr food deprivation. The authors should also compare HLH-30 localization between well-fed and 3hr FD animals to judge if 3hr FD increases cytoplasmic accumulation of HLH-30.

We have further analyzed the *hlh-30::gfp* reporter and found its dynamics to be complex, which can be seen in a new Figure 4H and Supplementary video 3. We see that HLH-30 localization becomes nuclear around one hour of food deprivation (Figure 4H and Figure 4G). Further, in a closer re-analysis and additional experiments, we do find that 1hr of food-deprivation is not sufficient to alter behavior in the sensory integration assay. Consistently, we see that 1hr food-deprived animals show reduced cytoplasmic localization and increased HLH-30::GFP nuclear localization (similar to the time course of the nucleus-to-cytoplasmic translocation for MML-1 that we have shown previously). Moreover, we find that 2 -3 hours of food-deprivation does indeed increase cytoplasmic *hlh-30::gfp* localization (Figure 4H and 4G, Supplementary Video 3). We include these details in our revised manuscript. Line 576-587.

6. To assess whether mml-1 and daf-2 function in the same pathway, the authors examined the phenotype of the double mutant of mml-1 and daf-2 and compared it with each single mutant (line 288-290, page 13; Fig. 5E). Because in both mml-1 and daf-2 mutants there is no significant difference between WF and FD animals in copper response (Fig. 5E), authors cannot claim that these are on the same pathway just by looking at double mutants. Assuming daf-2 and mml-1 are on different pathways, what would be the double mutant phenotype? Probably the same. Phenotypes of single mutants already show that both MML-1 and DAF-2 need to be intact for the starvation response to occur. In line 288, authors state "upstream MML-1 and downstream DAF-2", but this is not a genetically proven relationship, either. Therefore, if the authors want to claim more on the relationship between mml-1/hlh-30 and insulin pathway, they should test it in other ways. For example, fluorescence imaging experiment might be possible. As the authors suggested that expression of some insulin-like genes may be regulated by HLH-30, the authors could test insulin-like peptide expression using fluorescence reporter in several conditions, such as food deprivation and genetic background of mml-1 and hlh-30 mutants.

We agree with the reviewer and have removed panel 5E in our revised manuscript in the interest of clarity. However, we do find that *ins-31* in the intestine is sufficient to affect food-deprivation evoked behavioral change. We are unable to link *ins-31* directly to *mml-1*/*hlh-30* and so have toned down our conclusions in the revised manuscript. Line 629-781.

Minor points
1. Regarding the daf-28(sa191) mutant, the authors mentioned that this mutant is defective in multiple insulin-like peptides based on the previous findings (line265-267, page 12). This mutant was also shown to be defective in daf-7, TGFb, function (PLoS Genet. 2016 Dec 7;12(12):e1006450.). The authors could discuss about a possible function of signaling pathway other than insulin pathway, such as TGFb pathway, in copper response plasticity.

We thank the reviewer for pointing out this study. To reduce the complexity of our work and focus on the role of *ins-31*, we have removed the data referencing *daf-28*. We hope to address this in future publications.

2. In Supplemental table, three lines of rict-1 transgenic lines carrying Pstr-3::rict-1 are shown. But Fig. 6C shows results of only two of those transgenic lines.

To be completely transparent we included all transgenic lines that we tested. However, we have removed panel 6C in our revised manuscript in the interest of clarity. We plan on following up on this manuscript by probing the link between transcription factors in the intestine and specific neuronal circuits.

3. The authors show the possibility that PI3K and Rictor (RICT-1) may function in the same pathway in ASI neurons (line 310-311, page 14). To examine the genetic interactions between mTORC2 pathway and DAF-2/PI3K pathway, the authors could use sgk-1 mutants. SGK-1 was reported to function downstream of RICT-1 and there are a loss-of-function mutant, sgk-1(ok538) and a gain-of-function mutant, sgk-1(ft15), which are useful for epistasis analysis (Genes Dev 2016 Jul 1;30(13):1515-28).

We agree with the reviewer that it would be interesting to test the role of SGK-1 and appreciate the suggestion of the gain of function mutant. However, we have tried to simplify the paper and removed the section dealing with ASI neurons (as recommended by reviewer 2). We hope to further probe the role of non-canonical insulin signaling in neuronal activity.

**Reviewer #2:** Chalasani and colleagues report a study of altered risk-taking behavior in response to food deprivation, establishing a novel assay combining food deprivation with subsequent challenge to toxic copper and a food signal. Genetic analysis of established mutants and pathways are conducted to support a narrative involving the effect of food deprivation in modulating the transcriptional regulation of insulin ligands in the intestine, acting on the DAF-2 insulin receptor pathway in the ASI chemosensory neurons to modulate behavioral output. The study of gut-to-brain signaling is an area of wide interest, and the C. elegans system is well-suited to mechanistic dissection of molecular and cellular mechanisms and circuits involved in a simple animal system with tractable behavioral outputs. The manuscript is ambitious in tackling this question and touches upon all of the interesting relevant questions that might connect the observed behavioral system to the underlying molecular signaling.

Strengths of the manuscript include the careful analysis of behavior and the manner in which the authors address how to interpret the design of their behavioral assay, including control experiments that get at potential caveats and limitations. Important weakness in the manuscript that should be addressed are in the somewhat superficial genetic analysis. Behavioral genetics is challenging because the effect sizes are often not large, and thus appropriate rigor is needed if there is to be any sort of take-home message from the analysis.

We thank the reviewer for their positive feedback.

Some suggestions:
1. The authors conclude that food deprivation and not absence of chemosensory cues, account for the alterations during food deprivation. Beads are tested. What about heat-killed and/or aztreonam-treated bacteria?

For this resubmission, we designed an experiment to test the role of chemosensory cues. We placed bacteria on the lid of a plate and found that it was insufficient to alter food-deprivation evoked change. This assay uses agar plugs containing an OP50 lawn attached to the top of the lid of the starvation or well-fed plates. These data show that the odor of food is not sufficient to rescue the food deprivation behavior. These data are provided in Figure 3G and 3H.

2. Mml-1 effect is not a large one. Rescue experiments and/or multiple alleles should be examined.

We disagree that the effect of *mml-1* is not large; in the sensory integration assay *mml-1* mutant animals consistently do not change their behavior in response to food deprivation (chemotactic indices are ~0.4 in both well-fed and food-deprived animals, Figure 4B). Consistently, we show that *mml-1::gfp* leaves intestinal nuclei during a 3 hour food-deprivation (from ~25% of animals displaying nuclear localization to ~0% of animals showing nuclear localization in Figure 4D).

3. On the other hand, the hlh-30 shows a robust effect. As the story builds based on the rationale that HLH-30 may regulate insulin gene expression, rescue experiments and intestine-specific rescue in particular would strengthen the intestine-behavior link.\

We have attempted this experiment and have been unsuccessful. We agree that this would strengthen our manuscript and do intend to follow up on the link between transcription factors and insulin signaling in future studies.

4. Are the insulin ligands expressed in the intestine?

Ritter et al 2013 suggest that insulin ligands have complex expression patterns and are expressed in multiple tissues including the intestine. We cite these papers in our revised manuscript <https://pubmed.ncbi.nlm.nih.gov/34048707/> and <https://www.ncbi.nlm.nih.gov/pmc/articles/PMC6204898/>, which both show that *ins-31* has effects in hermaphrodites (Lines 1030-1050). Our understanding of the role for *ins-31* in the agonism and antagonism of *daf-2* is limited and likely complex. We do show that expression of *ins-31* cDNA is sufficient to rescue the behavioral phenotype, indicating that INS-31 from the intestine is critical to this mechanism (Figure 5D).

5. In the analysis of the insulin pathway (ligands and pathway components), even where statistical analysis supports the authors’ interpretation, there is a trend towards negative results. Particularly where conclusions are drawn, analysis of multiple alleles and rescue experiments are necessary—i.e. for ins-23, ins-31. For daf-28, lf alleles should probably be examined. The interpretation of the sa191 allele is complex (see Kulalert et al., 2013).

We completely agree with the reviewer and have tried to very transparent with our datasets. Our assay is based on a large number of animals and is variable. We added additional N to our insulin assays and we find that *ins-31* is the only consistent mutant that affects food-deprivation evoked behavioral change. Also, we are able to rescue this mutant in the intestine, but not neurons (Fig. 5D). For clarity, we have removed the data regarding *daf-28* and hope to pursue its complex role in future studies.

6. The genetic analysis of daf-2 is confusing (admittedly, it's always confusing). In one set of rescue experiments, the m596 allele is used, but ASI rescue is not done. In another set of experiments, all analysis is done in the mml-1; daf-2 (e1370) double mutant; each single has a phenotype. But instead of simply showing single mutant rescue, a somewhat convoluted double mutant rescue using tissue-specific promoters is shown. This should be clarified with corresponding rescue of single mutants. Also, the switching of alleles in the case of daf-2 is non-trivial given the many alleles of daf-2 and their altered strengths and activities.

In our attempt to link intestinal signaling with specific chemosensory neurons, we attempted to generate double rescues (rescuing *mml-1* in the intestine and *daf-2* in ASI chemosensory neurons). However, we agree with the reviewer that switching *daf-2* alleles will complicate our interpretation of the results. So, we have removed these data to streamline our revised manuscript. We plan on following on these experiments in future studies, which require the creation of many more *C. elegans* rescue lines in a variety of genetic backgrounds that lie outside the scope of this paper. Our data still includes both *daf-2* strains – *m596* and *e1370*, which both display similar chemotactic behaviors upon food deprivation (CI = ~0.5 for both well-fed and food-deprived, Figure 5E).

7. Rict-1 analysis shows small effects; multiple alleles and rescue not only with ASI promoter but also intestine and pan-neuronal promoters should be shown.

We have removed all data linking the signaling to ASI chemosensory neurons to streamline our revised manuscript.

The paper is ambitious in scope, and there is a lot of data in this paper. Where the essential basic amount of rigor cannot be provided, perhaps simply omitting these data/claims would also be a reasonable option.

We agree with the reviewer and have removed data to clarify our manuscript We have removed former Figures 5E showing *mml-;daf-2* double mutants and rescue and Figure 6C showing *rict-1* rescue in ASI. Specifically, we have focused our manuscript on signaling between the intestine and neurons and show that *ins-31* is rescued in the intestine, while *daf-2* can be rescued in neurons. We have removed all data linking the phenotype to ASI chemosensory neurons.

**Reviewer #3:** Matty et al. explore the modulation of chemosensory repulsion by feeding state through an impressive array of experiments that explore behavioral, sensory, and molecular dimensions of the phenomenon. The experiments are done to a high standard for the most part. However, the manuscript is a little confusing, and the broader conceptual framework needs to be reconsidered in my opinion.

Overall comments.
I question that the phenomenon under study here really has much to do with “integration”—which implies to me an interaction or dependency between two kinds of stimuli or information. Here, it is clear that starvation modulates response to the repellent copper, but irrespective of whether there is an attractive odor present. Sometimes “integration” seems to refer to the DA / Cu decision-making task (“sensory integration assay”), although these modalities seem to be independent of one another in their results, and sometimes to the effect of intestinal peptides on ASI. It’s not clear to me how a uni-modal interaction between the intestine and ASI constitutes “integration,” particularly in cases like line 98, where “integrate” is simply used as a transitive verb on a single object (“peptides”). I can’t see a cell responding to extracellular cues as integration, necessarily…it’s just responding to a signal. So this framework feels muddled… the authors need to be clearer about what they think “integration” means in the context of their experiments and be clear about which of the phenomena they describe constitute its mechanism. Their findings would be greatly strengthened by demonstrating an effect of starvation (and better yet, the putative peptide signals from the intestine in the absence of real starvation) on ASI neuronal function and/or circuit activity related to behavioral avoidance.

We thank the reviewer for bringing up an important point. We will continue to use the phrase “sensory integration assay” to refer to the assay we perform, for consistency between published work (Ishihara et al 2002). Elsewhere, we have revised our manuscript to limit using the word “integration” to refer to the function of neurons in responding to signals from the intestine. In other situation, we use repellent responses or sensory behavior as appropriate.

Specific comments
1. Previous research implicates ASH in being responsible for aversive responses to Cu, and inhibitory signaling between ASI and ASH. I’m unaware of studies showing that ASI plays a role in Cu avoidance behavior… where experiments done excluding ASH involvement? What led the authors to ASI? What circuit mechanisms plausibly explains ASI’s involvement?

We agree with the reviewer. ASH and ADL neurons have been shown to detect and respond to copper stimuli. Previous studies have shown that ASI and ASH neurons can reciprocally inhibit each other (Guo et al 2015). We include a comment about this interaction in our revised Discussion (Line 1125-1127).

2. Ghosh et al. showed that DA will induce animals to cross a hyperosmotic barrier, but no compelling effect is seen in Fig 1C – is this related to the low concentration of DA used here? A much higher concentration is used in Fig 1G.

Differences in glycerol concentrations and/or assay design might account for these differences. Our fructose concentrations range from 1-5 M in an individual line, while Ghosh et al 2016 use a ring of fructose. At 3 M, their data show that 1 and 5 hours of food deprivation increase hyperosmotic ring leaving. Our data, which is at 3 hours of food deprivation and 2.5M, actually show a significant, but slight increase in hyperosmotic barrier crossing. However, our data at 5M do not continue to show this change in behavior. We have included a comment about this in our revised manuscript. Line 255.

2. Figure 2C – the results here are non-significant, but it is doubtful that a significant differencts of the size shown could be detected given the sample size used. I don’t think any conclusion should be drawn form this, or the experiment should be repeated with samples appropriate to the effect size of interest.

As mentioned to reviewer 1, We have included additional data to show that food-deprived animals are more likely to cross the copper barrier. We were able to include additional data (N=5) to show that food-deprived animals cross the copper barrier in significant numbers compared to their well-fed counterparts in assays in which there is no attractant. We do note, however, that the proportion of animals crossing is still low and indicates that the use of an attractant makes these assays more robust. These data are presented in updated figures 2F, G, and H. We also included more N for the other conditions in this assay, leading to updated Figures 2C-E and Figures 2I-K. Further, we have simplified and clarified the methods for this tracking in Supplementary Figure S4A, and Supplementary Figure S4E-G.

3. Fig 5E and Fig 6C - last two groups in both have identically labelled x-axis categories

We have removed both panels 5E and 6C in our revised manuscript, deeming the proper experiments and analyses outside the scope of this current paper. Regarding the identical labels, we showed data from two independent transgenic animals in our initial draft.

4. Fig 5. Are age-1 animals sickly or movement impaired? Difficult to conclude anything about the effects of FD if they are generally poor at chemotaxis.

We show that *age-1* mutants are able to chemotax towards diacetyl without a copper barrier (particularly food-deprived animals) confirming that they are able to move (Supplementary Table 1) . We include a comment in our revised manuscript with a reference to describe the chemotactic index when no copper barrier is present (for mutant strains with particularly low chemotactic indices). These results suggest that while *age-1* mutants can move, they are defective in the food-deprivation evoked change in sensory integration behavior. Line 934-936.
