# Supplementary material for: Intestine-to-neuronal signaling alters risk-taking behaviors in food-deprived Caenorhabditis elegans
Source: PLoS Genet. 2022 May 5;18(5):e1010178. doi: 10.1371/journal.pgen.1010178 (PMC9070953; doi:10.1371/journal.pgen.1010178)
Supplement: Attachment — Submitted filename: Matty_revisionresponses.docx [file pgen.1010178.s011.docx]

Responses (in black) to reviewers comments (in blue)

Reviewer #2: The revised manuscript has been improved by the removal of data that are not as well substantiated. The narrative of intestine-to-neuron signaling would benefit from strengthening the rigor of the genetic analysis, as suggested in the initial review. I repeat basic suggestions from my initial review:

*We thank the reviewer for their continued attention to detail and interest in our manuscript. We hope that our comments and manuscript edits address your concerns.*

1) Heat-killed and/or aztreonam-treated bacteria can not only assess for the effect of volatile cues, but also water-soluble cues, which are not assayed in the revision.
   a. *We appreciate this comment and have been more specific in the manuscript about the types of odors our new experiment includes. Please see manuscript lines 231-236, where we refer to these agar-plug OP50-associated odors as volatile.*
   b. *Further, we would wholeheartedly expect animals places on heat-killed bacteria to behave as though they are well-fed, but those experiments have not yet been performed in our lab. We are interested in the role of actively metabolizing bacteria as members of the gut microbiota and anticipate investigating this in the future.*
   c. *In our hands following existing protocols, aztreonam-treated bacteria often grow to sizes that are inedible to C. elegans, but there remain smaller bacteria or broken bacterial segments that remain edible in the mixture. We have performed these experiments with mixed results, and were unable to convince ourselves that the C. elegans were not consuming at least some bacteria.*
2) The narrative argues strongly for a role for MML-1 and HLH-30 in the intestine. Rescue experiments and/or multiple alleles represent a minimum level of genetic rigor to make the point.
   a. *We appreciate this point and also strive for genetic rigor. At this time, there is only one available/published allele each for MML-1 and HLH-30.*
   b. *Without these the production, validation, and use of novel genetic resources, we have added a clarification in lines 384-386, stating that it is possible that ins-31 is uncoupled from MML-1 and HLH-30.*
3) The ins-31 intestine rescue experiments have been performed, but the data in Figure 5D are not convincing. Why does the mutant data look so different from the data with the presumably nonfunctional neuronally-expressed ins-31?
   a. *We appreciate this point and understand that ectopic, exogenous expression of insulin cDNA might disrupt neuronal function in a manner that affects chemotaxis behavior. We have added this interpretation to lines 317-319.*
